# Activin A and Cell-Surface GRP78 Are Novel Targetable RhoA Activators for Diabetic Kidney Disease

**DOI:** 10.3390/ijms22062839

**Published:** 2021-03-11

**Authors:** Asfia Soomro, Jackie Trink, Kian O’Neil, Renzhong Li, Safaa Naiel, Bo Gao, Kjetil Ask, Joan C. Krepinsky

**Affiliations:** 1 Division of Nephrology, Department of Medicine, McMaster University, Hamilton, ON L8N 4A6, Canada; soomroa@mcmaster.ca (A.S.); trinkj1@mcmaster.ca (J.T.); oneilk9@mcmaster.ca (K.O.); lirenz@mcmaster.ca (R.L.); gaolinbo@hotmail.com (B.G.); 2 Division of Respirology, Department of Medicine, McMaster University, Hamilton, ON L8N 4A6, Canada; naiels@mcmaster.ca (S.N.); askkj@mcmaster.ca (K.A.)

**Keywords:** diabetic kidney disease, RhoA, Rho-kinase, fibrosis, activins, cell-surface GRP78

## Abstract

Diabetic kidney disease (DKD) is the leading cause of kidney failure. RhoA/Rho-associated protein kinase (ROCK) signaling is a recognized mediator of its pathogenesis, largely through mediating the profibrotic response. While RhoA activation is not feasible due to the central role it plays in normal physiology, ROCK inhibition has been found to be effective in attenuating DKD in preclinical models. However, this has not been evaluated in clinical studies as of yet. Alternate means of inhibiting RhoA/ROCK signaling involve the identification of disease-specific activators. This report presents evidence showing the activation of RhoA/ROCK signaling both in vitro in glomerular mesangial cells and in vivo in diabetic kidneys by two recently described novel pathogenic mediators of fibrosis in DKD, activins and cell-surface GRP78. Neither are present in normal kidneys. Activin inhibition with follistatin and neutralization of cell-surface GRP78 using a specific antibody blocked RhoA activation in mesangial cells and in diabetic kidneys. These data identify two novel RhoA/ROCK activators in diabetic kidneys that can be evaluated for their efficacy in inhibiting the progression of DKD.

## 1. Introduction

Compared to all other etiologies of progressive kidney disease, diabetic kidney disease (DKD) is the leading cause of morbidity and mortality in developed countries, placing a substantial economic burden on healthcare systems. Current treatment options, including glucose and blood pressure control, use of renin-angiotensin system blockers and, more recently, use of SGLT2 (sodium-glucose cotransporter-2) inhibitors in those with type 2 diabetes, slow disease progression. However, DKD remains the leading cause of kidney failure globally, accounting for almost half of those with end-stage kidney disease requiring dialysis or transplantation [1,2]. A continued improvement in our understanding of the molecular mechanisms underlying its pathogenesis will enable the development of additional complementary therapeutics that will impact DKD progression.

Although pathological changes in DKD ultimately occur in all kidney compartments, glomerular basement membrane thickening and mesangial matrix expansion are the hallmark and earliest manifestations, with the latter due to the increased production of extracellular matrix (ECM) proteins by mesangial cells (MC) [3,4]. RhoA/Rho-associated protein kinase (ROCK) activation has been demonstrated in MC, as well as other glomerular and tubulointerstitial cells, in response to various stimuli relevant to DKD. These include high glucose (HG), growth factors, the profibrotic cytokine transforming growth factor β1 (TGFβ1) and mechanical signals [5,6,7,8]. Indeed, RhoA appears to be a central mediator and integrator of several profibrotic stimuli relevant to diabetes.

RhoA belongs to the Rho family of small GTPases, cycling between an active GTP-bound and an inactive GDP-bound form. A conformational change induced by GTP binding allows RhoA to interact with and activate downstream proteins by relieving intramolecular autoinhibitory interactions, with the serine–threonine ROCK, a major downstream mediator, having the most established relevance to DKD [9,10]. Through its role as a regulator of cytoskeletal dynamics, as well as its modulation of profibrotic gene expression more directly, RhoA signaling has been shown to regulate the activation of profibrotic signaling pathways and thus the production of ECM [5,10].

The importance of ROCK in DKD has been shown in vivo through pharmacologic or genetic inhibition studies in diabetic mice or rats, with inhibition reducing albuminuria, mesangial expansion, and ECM production [10]. While these studies have suggested for some time that ROCK may be a potential therapeutic target for DKD, there are as yet no clinical trials to evaluate its efficacy despite its clinical evaluation for other disorders [10]. This may be due to its renal hemodynamic effects, particularly in diabetics, which are possibly accentuated when used in combination with inhibitors of the renin–angiotensin system [11]. Similarly, direct inhibition of RhoA is unlikely to be feasible given its importance in normal cellular physiology. A potential alternative approach may be to target distinct activators of RhoA signaling that are selective for DKD.

Recently, two novel mediators of the pathogenesis of DKD have emerged. These are the upregulation of, and signaling by, TGFβ superfamily members known as activins, as well as the HG-induced translocation of the intracellular protein GRP78 to the cell surface to act as an initiator of profibrotic signaling. Both are significantly activated in DKD, with a minimal presence in normal kidneys [12,13]. Whether they integrate with RhoA signaling, however, is unknown. Therefore, in this study, we assess their ability to activate RhoA/ROCK in cultured MC and in DKD. We propose that an approach to diminishing renal fibrosis via RhoA/ROCK attenuation is in the single or combined inhibition of these novel targets.

## 2. Materials and Methods

### 2.1. Cell Culture

Primary mouse MC were isolated as described previously [14] and cultured in Dulbecco’s modified Eagle medium supplemented with 20% fetal bovine serum, streptomycin (100 μg/mL) and penicillin (100 μg/mL). They were grown at 37 °C in 95% O_2_ and 5% CO_2_. MC were serum deprived in medium with 1% bovine serum albumin prior to treatment.

### 2.2. Protein Extraction and Immunoblotting

Whole-cell lysates were collected as previously described [8]. For biotinylation experiments, MC were incubated with 1 mg/mL EZ-linked Sulfo-Biotin (Pierce) for 30 min followed by 0.1 M glycine in PBS to remove excess Sulfo-Biotin, then whole-cell lysates were collected and incubated overnight in a 50% NeutrAvidin slurry (Fisher) to pull down biotin-tagged proteins. Immunoprecipitates were assessed with SDS–PAGE and immunoblotted for surface proteins. The following antibodies were used for immunoblotting: α-smooth muscle actin (αSMA) (Pierce, 1:5000), phosphorylated myosin phosphatase target (pMYPT) Thr696 (Cell Signaling; 1:1000), fibronectin (FN) (BD Biosciences, 1:1000), total RhoA (Santa Cruz, 1:500), MTJ-1 (Santa Cruz, 1:1000), GRP78 (BD Biosciences, 1:1000), PDGFRβ (Cedarlane, 1:1000), vinculin (Sigma, 1:1000) and tubulin (Santa Cruz, 1:5000).

### 2.3. RhoA Activity

Active RhoA was immunoprecipitated from freshly lysed cells using GST-tagged Rhotekin RBD (Upstate), which binds specifically to GTP-bound RhoA. To detect active, GTP-bound RhoA in kidneys, immunohistochemistry was conducted on paraffin embedded kidney sections (4 µm) using the conformation-sensitive RhoA antibody (New East Biosciences, 1:275) after heat-induced epitope retrieval (30 min steaming in citric acid). Twenty images were then taken at 60× for each section, quantified using Image J software, and values averaged to produce one value per image. This value was used for statistical analysis as described below.

### 2.4. Animal Studies

All studies were conducted in accordance with McMaster University and the Canadian Council on Animal Care guidelines. Male C57BL/6-InsAkita/J (Jackson Labs) type 1 diabetic mice and their wild-type controls were injected intraperitoneally (IP) with follistatin (3 µg) or the vehicle every other day from 6 to 18 weeks, as previously described [13]. Male CD1 mice (Charles River) underwent left nephrectomy at 9 weeks, followed one week later by IP injection of 200 mg/kg streptozotocin to induce type 1 diabetes. Control mice received an equal volume of citrate buffer. Mice confirmed to be diabetic were treated IP with the cell-surface GRP78-neutralizing antibody C38 (generously provided by S. Pizzo [15]) or isotype IgG2b (BioXCell) at an initial dose of 200 µg followed by 100µg weekly for a total of 12 weeks. Formalin-fixed tissue was used for immunohistochemistry studies.

### 2.5. Transfection, Luciferase and siRNA

MC were plated at 60–70% confluence and transfected with 0.5 μg of the alpha smooth muscle actin promoter (αSMA) luciferase reporter construct (pGal3-α-SMAp-luc), a generous gift from Dr. A. Kapus and 0.05 μg pCMV β-galactosidase (Clontech) using Effectene (Qiagen). After the cells were harvested, luciferase and β-galactosidase activities were measured using kits from Promega. For siRNA transfection, MC were plated at 50% confluence and transfected with 100 nM MTJ-1 siRNA (Thermo Fisher). The following day, cells were serum deprived prior to treatment.

### 2.6. RNA Extraction and qPCR

RNA was extracted from flash-frozen kidney sections using the RNeasy Plus Mini Kit (Qiagen). RNA was reverse transcribed using qScript Supermix Reagent (Quanta Biosciences) and quantitative real time PCR was performed using the Power SYBR Green PCR Master Mix on the Applied Biosystems Vii 7 Real-Time PCR System. Primer sequences used were as follows: CHOP forward 5′-GGAAACAGAGTGGTCATTCCC-3′ and reverse 5′-CTGCTTGAGCCGTTCATTCTC-3′ and IRE1α forward 5′-CCATCGAGCTGTGTGCAG-3′ and reverse 5′-TGTTGAGGGAGTGGAGGTG-3′. mRNA expression levels were determined relative to 18S of the same sample using the ΔΔCT method.

### 2.7. Statistical Analysis

One-way ANOVA was used for analysis, with Tukey’s HSD used for post hoc testing and *p* < 0.05 considered significant. Data were presented as mean ± SEM.

## 3. Results

### 3.1. RhoA Mediates the Profibrotic Effects of Activins in DKD

Activins are emerging as important mediators in the pathogenesis of DKD [16]. These members of the transforming growth factor β1 (TGFβ1) superfamily are glycoprotein dimers composed of two β-subunits of inhibin linked by disulfide bonds. Five isoforms have been identified in mammals, including activins A, B, AB, C, and E, with activin A being the most well studied to date. Activin A is important during kidney development, but is not present in the normal adult kidney. Activin B is also not normally present in adult kidneys, but its role in development and disease is as yet relatively unknown, with little information available for the other isoforms [17,18]. Analogous to TGFβ1, activins A and B signal through a complex of type I and II receptors to activate Smad3, with consequent regulation of gene expression through the Smad-binding element [17,18]. Interestingly, in various cells, including renal fibroblasts and MC, activins have also been shown to be critical for the profibrotic response to TGFβ1 [14,19,20,21], a cytokine central to the pathogenesis of DKD [22], although the mechanism underlying this has not been defined as of yet. We and others have recently shown activin A to be induced in DKD [13,23] and to be a prominent mediator of HG-induced profibrotic responses in MC. We have also recently observed an increase in activin B in DKD (unpublished). Importantly, the activin inhibitor follistatin improved DKD in type 1 diabetic Akita mice [13].

**Figure 1:**
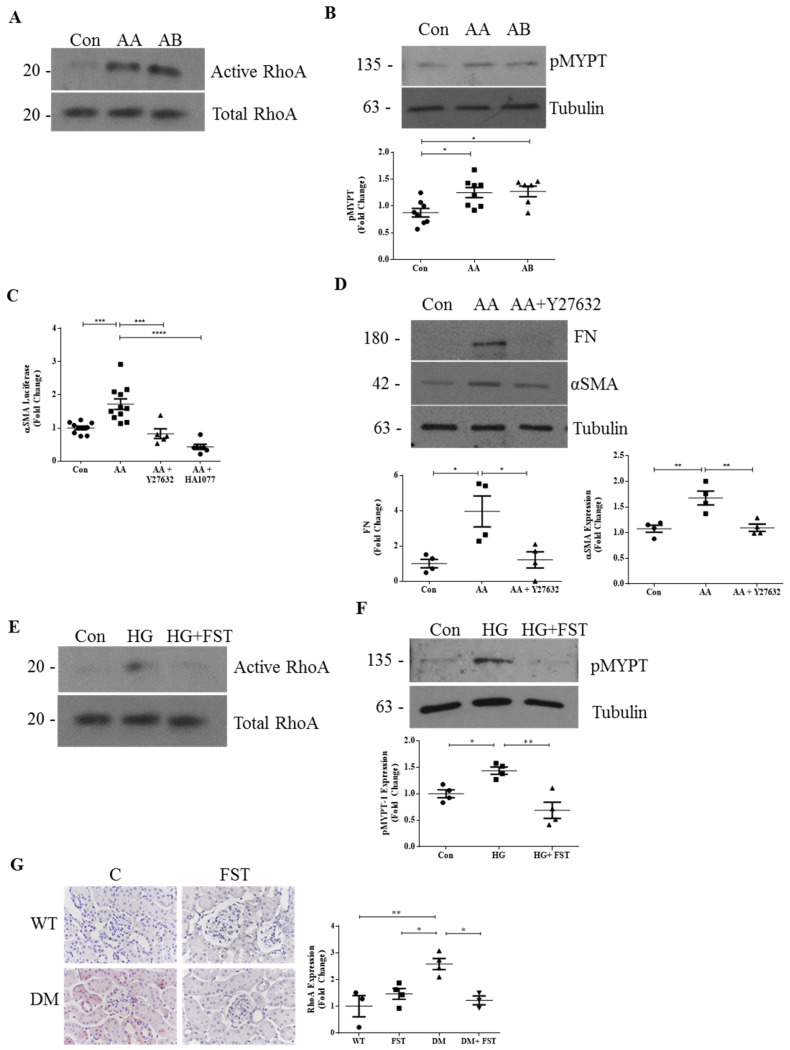
RhoA mediates the profibrotic effects of activins in diabetic kidney disease (DKD). (**A**) Activin A (20 ng/mL, 3 h) and activin B (20 ng/mL, 3 h) both activated RhoA in mesangial cells (MC) (*n* = 2). (**B**) Activation of downstream Rho-kinase was also seen, assessed by phosphorylation of myosin phosphatase target (MYPT) (pMYPT Thr696) in MC (*n* = 6–8 * *p* < 0.05). (**C**) Rho-kinase inhibition with Y27632 (10 μM) or HA1077 (10μM) prevented activin A (20 ng/mL)-induced α-smooth muscle actin (αSMA) promoter luciferase activation (*n* = 6–11 *** *p* < 0.001, **** *p* < 0.0001) and (**D**) protein expression of both αSMA and the fibrotic protein fibronectin (FN) (*n* = 4, * *p* < 0.05, ** *p* < 0.01). (**E**) The activin inhibitor follistatin (FST, 500 ng/mL) prevented high glucose (HG) (3 h)-induced RhoA activation (*n* = 2) and (**F**) Rho-kinase activation (*n* = 3, * *p* < 0.05, ** *p* < 0.01). (**G**) RhoA activation in kidneys was also significantly inhibited by follistatin in type 1 diabetic Akita mice, as assessed by IHC with an antibody which specifically detects active RhoA (*n* = 4, * *p* < 0.05, ** *p* < 0.01).

While the activation of RhoA/Rho-kinase by HG in MC and in DKD has been well established [5,10], whether activins are needed for this is as yet unknown. In non-malignant cells, activin B was shown to induce RhoA activation [24,25,26]. Whether activin A similarly induces its activation and whether this occurs in MC have not been determined as of yet. We therefore tested both activins for their ability to induce RhoA signaling. Figure 1A,B show that both activins induced RhoA and downstream ROCK activation, with the latter assessed by phosphorylation of its target protein myosin phosphatase target subunit 1 (pMYPT1) at Thr696. Since increased expression of α-smooth muscle actin (αSMA) is a characteristic of activated MC, which synthesize matrix proteins [27], and RhoA/ROCK signaling is known to regulate αSMA transcription [28], we next tested the ability of activins to increase αSMA production in MC. Figure 1C,D show that activin A induced αSMA promoter activation and protein expression in MC, and that this was prevented by ROCK inhibition with either Y27632 or HA1077. We further confirmed that activin A-induced upregulation of the profibrotic protein fibronectin was also attenuated by Y27632 (Figure 1D).

These data suggested that RhoA signaling may mediate activin profibrotic responses in DKD. This was tested using the activin inhibitor follistatin. As seen in Figure 1E,F, follistatin effectively blocked HG-induced RhoA and downstream ROCK activation. As we have shown that follistatin inhibits DKD in type 1 diabetic Akita mice without affecting blood glucose levels [13], we next assessed the relevance of these findings in vivo. Using an antibody specific to active RhoA, immunohistochemistry confirmed that activation of RhoA, which is increased in the kidneys of diabetic mice, was attenuated by treatment with follistatin (Figure 1G). Taken together, these data indicate that RhoA is an important mediator of activin responses both in vitro and in vivo.

### 3.2. Cell-Surface GRP78 Mediates HG-Induced RhoA Activation

We have recently identified cell-surface (cs)GRP78 as a novel pathologic signaling receptor in DKD [12]. Endogenously, GRP78 is well known as an endoplasmic reticulum (ER) chaperone responsible for proper protein folding and homeostasis. More recently, GRP78 has also been shown to translocate to the surface of various cell types under conditions of ER stress. While this has been best described in cancer cells, we have also shown this to occur in response to HG in MC and in glomeruli from diabetic mice [12,29,30]. Once at the cell surface, GRP78 promotes profibrotic PI3K/Akt activation to potentiate the accumulation of ECM proteins in MC exposed to HG [12]. Recently, RhoA/ROCK-mediated PI3K/Akt activation via csGRP78 has been shown in pancreatic cancer cells [31]. We were therefore interested in determining whether RhoA signaling was also activated downstream of csGRP78 in response to HG.

**Figure 2:**
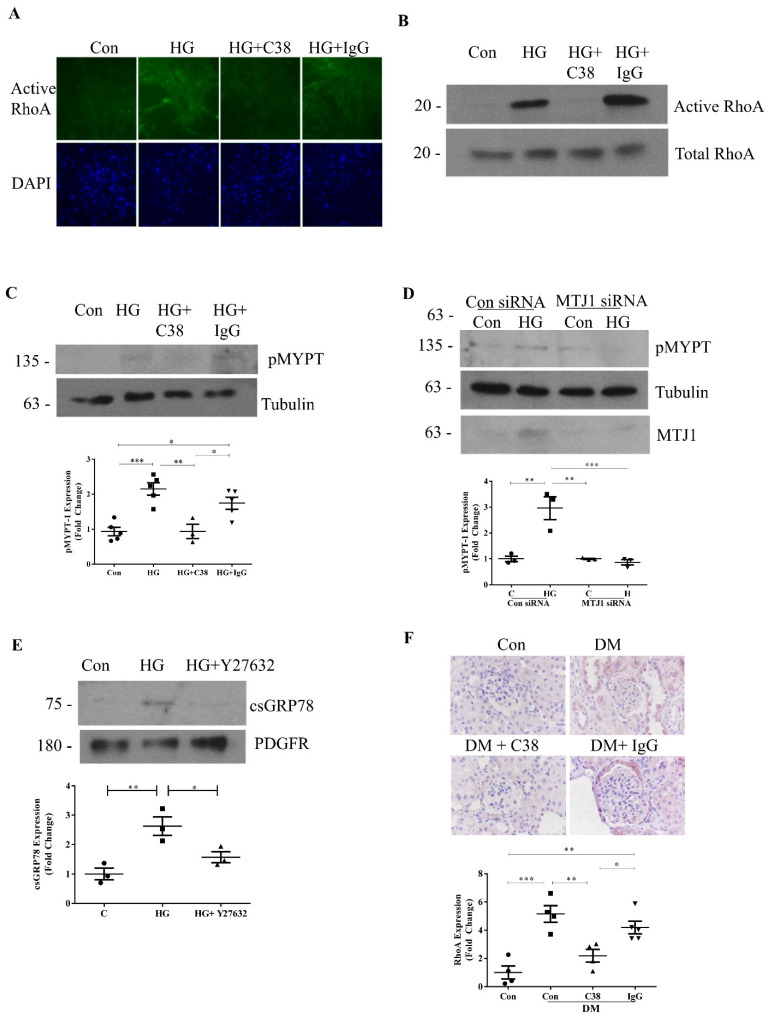
Cell-surface GRP78 mediates HG-induced RhoA activation. Inhibition of cell-surface GRP78 with an antibody specific to its C-terminus (C38, 2 µg), but not control IgG, attenuated HG-induced RhoA activation, assessed by immunofluorescence using an active RhoA-specific antibody (**A**) and pull-down and immunoblotting of active RhoA (**B**). (**C**) Immunoblotting confirmed inhibition of HG-induced Rho-kinase activation by C38, assessed by phosphorylation of MYPT (*n* = 5, * *p* < 0.05, ** *p* < 0.01, *** *p* < 0.001). (**D**) siRNA knockdown of MTJ-1 to prevent GRP78 localization to the cell-surface also inhibited Rho-kinase activation by HG (*n* = 3, ** *p* < 0.01, *** *p* < 0.001). (**E**) Conversely, HG (48 h)-induced cell-surface GRP78 localization was attenuated by Rho-kinase inhibition with Y27632 (10μM) (*n* = 3, * *p* < 0.05, ** *p* < 0.01). (**F**) RhoA activation was assessed by immunohistochemistry in kidneys of uninephrectomized CD1 mice made diabetic by streptozotocin. Activation was significantly inhibited by cell-surface GRP78 inhibition with C38 compared to control IgG (*n* = 4, * *p* < 0.05, ** *p* < 0.01, *** *p* < 0.001).

We and others previously showed that cell-surface GRP78 signaling can be inhibited with antibodies that target its C terminus [12,15]. We thus tested the effects of a C-terminus-targeting antibody (C38) on HG-induced RhoA activation. In Figure 2A, immunofluorescence using an antibody specific to active RhoA shows that C38, but not control IgG, inhibited HG-induced RhoA activation. This was confirmed using an assay to immunoprecipitate active RhoA (Figure 2B). Similar inhibition of HG-induced ROCK activation by C38 was seen, assessed by phosphorylation of MYPT (Figure 2C). Downregulation by siRNA of MTJ-1, a co-chaperone required for cell-surface translocation of GRP78 [32], further supported a role for csGRP78 in HG-induced ROCK activation (Figure 2D). Given the important role of RhoA/ROCK in cytoskeletal reorganization and intracellular trafficking [33], we further assessed whether the movement of GRP78 to the cell surface in response to longer-term HG exposure could also be regulated by RhoA signaling. Interestingly, Figure 2E shows that HG-induced csGRP78 localization, as assessed by the biotinylation of cell-surface proteins followed by immunoblotting for GRP78, was prevented by the ROCK inhibitor Y27632. These data implicate a bidirectional dependence of csGRP78 and RhoA in HG, whereby csGRP78 both serves as an upstream regulator of RhoA activity, and also requires its signaling for movement to the cell surface.

**Figure A1:**
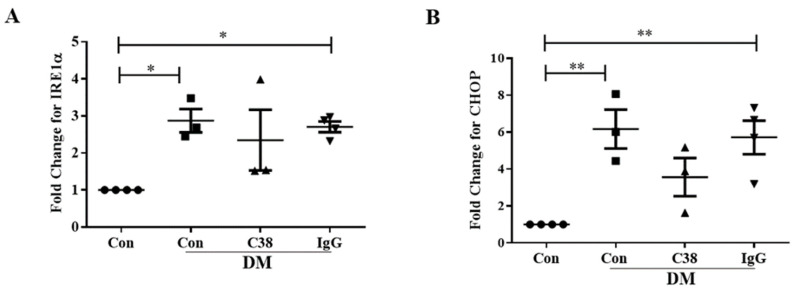
Cell surface GRP78 inhibition does not attenuate ER stress. Transcript levels for the ER stress response genes IRE1α (**A**) and CHOP (**B**) were assessed by qPCR. The increase seen in diabetic mice was not attenuated by treatment with C38 (*n* = 4, * *p* < 0.05, ** *p* < 0.01).

Finally, to test the in vivo relevance of this model, we assessed RhoA activity by immunohistochemistry in type 1 diabetic CD1 mice treated with the C38 antibody, as described in the Materials and Methods. Treatment did not affect glucose levels (con 10.1 ± 1.4 mM, DM 32.9 ± 0.3 mM, DM + C38 31.6 ± 0.9 mM, DM + IgG 29.3 ± 0.8 mM). Figure 2F shows that csGRP78 inhibition, but not the isotype IgG, attenuated RhoA activation in diabetic kidneys. Taken together, RhoA/ROCK signaling is also a downstream mediator of csGRP78-induced profibrotic responses, likely through regulation of PI3K/Akt signaling [12,31]. Finally, given the involvement of GRP78 in ER stress, we further assessed if the attenuation of RhoA activation observed with the C38 antibody was also associated with a reduction in ER stress. We assessed the transcript levels of well-established ER stress response genes, CHOP and IRE1α [29]. As shown in Appendix A Figure A1, these were significantly increased in diabetic mice, but were unaffected by treatment with C38, suggesting that inhibition of csGRP78 does not affect ER stress in this model.

## 4. Discussion and Conclusions

DKD is the leading cause of end-stage kidney disease. Despite the significant recent advancement in treatment options with the advent of SGLT2 inhibitors, ongoing efforts to develop additive therapies that can prevent the progression of DKD are needed. The central importance of RhoA/ROCK signaling in the pathogenesis of DKD has been well established in cell culture studies and animal models. However, this has not as yet translated into clinical practice. RhoA appears to integrate numerous profibrotic signals relevant to the diabetic state and, in this study, we show that signaling through both activins and csGRP78 also relies on RhoA/ROCK activation. Importantly, we show that their inhibition in vivo efficiently suppresses RhoA activation in diabetic kidneys, suggesting that their targeting may be a novel and effective means of inhibiting the progression of DKD.

Two ROCK isoforms exist, but a greater understanding is needed with regard to their individual roles in DKD. In cell culture, differential effects of these isoforms have been observed in different cells. For example, ROCK2, but not ROCK1, deletion inhibited TGFβ1-induced profibrotic responses in MC [34]. Conversely, ROCK1 deletion attenuated HG-induced apoptosis in podocytes and endothelial cells [35]. Thus, it would appear that inhibition of both isoforms may be needed for DKD protection. Indeed, the nonselective ROCK inhibitor fasudil protected against DKD in both type 1 and 2 diabetic models [5,36,37], although it is important to note that it may inhibit other serine/threonine kinases at similar or higher concentrations [38]. However, knockout or inhibition of individual isoforms has also shown overall in vivo benefits. ROCK1 knockout mice had decreased albuminuria, mesangial matrix expansion, glomerular basement membrane thickness and loss of podocytes in both type 1 (streptozotocin-induced) and 2 (db/db) diabetic mice [39,40]. The recently developed selective ROCK2 inhibitor KD025 was also shown to attenuate albuminuria and glomerular fibrosis in db/db mice [35]. It thus appears that in vivo inhibition of either isoform provides benefits. However, ROCK is important to the renal regulation of blood flow, particularly in diabetic animals [11]. While it is not known whether this is isoform specific as of yet, it remains to be seen whether ROCK inhibitors, in clinical practice, could safely be used and combined with inhibitors of the renin–angiotensin system or SGLT2 inhibitors that also have glomerular hemodynamic effects.

The direct targeting of RhoA is unlikely to be feasible given the important role of this GTPase in fundamental cellular processes. Indeed, RhoA knockout is embryonically lethal in mice [9]. Furthermore, podocyte-specific RhoA activation and inhibition both caused podocyte injury [41], illustrating the requirement for a fine balance in the activity of this pathway. Furthermore, there are several RhoA effectors other than ROCK, such as protein kinase N (PKN) and mDia, the roles of which have not as yet been investigated in DKD [9]. Indeed, PKN is closely related to protein kinase C, which has a well-established pathogenic role in DKD [42]. We thus propose that targeting other regulatory factors activating RhoA signaling may better modulate its activity to reduce pathologic activation while maintaining its ability to function in homeostasis.

An important activator of RhoA/ROCK is the profibrotic cytokine TGFβ1, a major and well recognized mediator of DKD [7,42,43]. Attempts have been made to target TGFβ clinically. However, it too has important functions which lead to adverse effects when inhibited. When used at tolerated doses, a recent clinical trial showed that a TGFβ1-neutralizing antibody was not clinically effective at slowing DKD, likely due to incomplete TGFβ1 neutralization in an effort to limit adverse effects [44]. Recently, activins have emerged as potentially important profibrotic mediators in DKD and other renal diseases [13,16]. Interestingly, although activins A and B signal through the same Smad mediators as TGFβ1, studies in various renal and other cells have shown that the profibrotic effect of TGFβ1 is dependent on the presence of activins [14,19,20,21]. The mechanism underlying this is not as yet known, but given that the expression of activins in normal adult kidneys is very low or absent, with significant upregulation in DKD, these cytokines offer an alternative therapeutic target for TGFβ1/RhoA inhibition.

Previous studies have shown that activin B activates RhoA/ROCK to regulate cell movement, cytoskeletal reorganization and wound healing [24,25,45]. In this study, we showed that activins A and B both directly regulate RhoA and downstream ROCK activity. Furthermore, their inhibition with follistatin, which we have previously shown to ameliorate DKD in type 1 diabetic mice [13], also inhibits both HG-induced RhoA/ROCK activation in MC and RhoA activation in diabetic kidneys. These data suggest that activins may mediate profibrotic responses through RhoA/ROCK signaling. Future studies are needed to investigate the crosstalk between Smad and RhoA/ROCK signaling by activins in the diabetic setting.

We have recently identified csGRP78 as an additional profibrotic mediator in DKD, the expression of which is absent in normal kidneys [12]. Thus, akin to activin expression in diabetic kidneys, csGRP78 may provide an alternative disease-specific target. In this study, we identify the involvement of csGRP78 in RhoA/ROCK activation by HG in MC and in diabetic kidneys. The activation of RhoA/ROCK by csGRP78 has also recently been shown in pancreatic cancer cells, where it mediated downstream PI3K/Akt activation [46]. Since we have previously shown that csGRP78 is required for PI3K/Akt activation, a known profibrotic pathway in response to HG [12,47], it is likely that RhoA/ROCK signaling lies upstream of this. Interestingly, cell-surface expression of GRP78 was also significantly reduced by ROCK inhibition, suggesting that RhoA/ROCK activation promotes further translocation of GRP78 to the cell surface. The regulation of cellular trafficking through cytoskeletal changes by RhoA [33] is likely to be important for these observations, but this needs to be confirmed experimentally. Alternatively, ROCK inhibition was shown to downregulate the expression of cellular GRP78 in human pulmonary microvascular endothelial cells [48]. A reduction in the total pool of GRP78 may lead to reduced cell-surface expression, although we have not observed any effects of HG on total GRP78 expression [12]. Altogether, this suggests an important role for csGRP78 in mediating RhoA/ROCK signaling as an important regulator of DKD pathogenesis.

In conclusion, our data identify two potential novel alternative approaches to targeting RhoA/ROCK-dependent profibrotic signaling in DKD. Here, both activins A/B and csGRP78 are viable potential candidates for anti-fibrotic therapeutics. Considering the important role of RhoA/ROCK in normal cellular function, these targets are attractive given their relative absence in normal kidneys. Indeed, several options are available and can be evaluated for targeting activins in DKD, including follistatin, ligand traps and neutralizing antibodies [16]. Of these, follistatin was shown to reduce DKD in a preclinical model [13], and some, such as ligand traps and neutralizing antibodies, are already in clinical development for other disorders [16]. Whether targeting csGRP78 in vivo benefits DKD must be tested, and studies using the C38 antibody are ongoing in our lab. A greater understanding of the mechanism by which csGRP78 is activated by HG would also assist in developing novel therapeutics in order to target this particular protein.

## Data Availability

Data are contained within the article and Appendix A.
